# Estimation of a Structural Equation Modeling of Quality of Life Mediated by Difficulty in Daily Life in Survivors of Breast Cancer

**DOI:** 10.3390/healthcare11142082

**Published:** 2023-07-21

**Authors:** Aki Watanabe, Takayuki Kawaguchi, Ayumi Nobematsu, Satoshi Sasada, Nozomi Kanari, Tatsuya Maru, Takeshi Kobayashi

**Affiliations:** 1 Faculty of Health and Social Work, School of Rehabilitation, Kanagawa University of Human Services, Yokosuka City 238-8522, Kanagawa, Japan; sasada@kuhs.ac.jp; 2 Department of Community Mental Health and Law, National Institute of Mental Health, National Center of Neurology and Psychiatry, Kodaira City 187-8553, Tokyo, Japan; kawagu-t@ncnp.go.jp; 3 Department of Rehabilitation, School of Allied Health Sciences, Kitasato University, Sagamihara City 252-0373, Kanagawa, Japan; ayumi.mnb017@gmail.com; 4 Department of Rehabilitation, Faculty of Health Sciences, Nihon Institute of Medical Science, Iruma County 350-0435, Saitama, Japan; n-kanari@nims.ac.jp (N.K.); maru@nims.ac.jp (T.M.); take-1008@jcom.home.ne.jp (T.K.)

**Keywords:** breast cancer, survivors, Bayesian structural equation modeling, quality of life, difficulty in daily life

## Abstract

Background: The purpose of this study was to clarify the structural relationship of quality of life (QOL) in survivors of breast cancer, including difficulty in daily life and negative experiences in daily activities, as health-related indicators. Methods: Participants were survivors of breast cancer for more than 2 years after primary breast cancer surgery and belonged to self-help groups. The assessment used FACT-B (QOL), HADS (anxiety and depression), SOC (sense of coherence), WHODAS 2.0 (difficulties in daily life), and CAOD (negative experiences in daily activities). Bayesian structural equation modeling (BSEM) was performed to analyze the hypothesized model. If the causal model was significant, multiplication of the path coefficient from emotional distress (anxiety and depression) to QOL, and from SOC to emotional distress, was considered a direct effect on QOL, and from SOC to difficulty in daily life, from difficulty in daily life to negative experiences in daily activities, and from negative experiences in daily activities to anxiety and depression were considered indirect effects on QOL. Results: The participants comprised 73 survivors of breast cancer. The goodness of fit of the model in the BSEM was satisfactory. The direct effect was 0.274, and the indirect effect was 0.164. Conclusions: An additional finding of this study is that coping with difficulty in daily life and negative experiences in daily activities related to QOL may improve QOL.

## 1. Introduction

Survivors of breast cancer are prone to experience psychological problems, so it is important to consider specific methods to improve their quality of life (QOL). This is due to the fact that many breast cancer patients have high survival rates after treatment and live for a long time as survivors. Psychological problems such as anxiety, depression, and poor body image are found in 20–40% of survivors of breast cancer 1 year after surgery and in 15% 5 years after diagnosis [1], and QOL is likely to decrease due to increased difficulties in daily life caused by treatment side effects and unemployment [2]. A literature search of electronic bibliography studies in 2023 [3] shows that several references indicate that patients with anxiety and depression have a poor QOL and a high need for supportive care. To maintain and improve QOL, it is important to improve these survivors’ sense of coherence (SOC) [4]. SOC is the ability to cope with stress and consists of three senses: “comprehensibility”, “manageability”, and “meaningfulness” [5]. “Comprehensibility” refers to the sense of being able to understand one’s current situation and predict future situations to some extent; “manageability” is the sense of being able to manage and get by in life; and “meaningfulness” is the sense of coping with stress and finding meaning in daily activities. SOC is a predictor of QOL [6] and using more coping strategies increases SOC [7]. It has also been reported that the higher the SOC, the lower the anxiety and depression, and the higher the QOL [8]. Rohani et al. [6] stated that, in patients with breast cancer, SOC is a main variable in the psychological process during the disease. In this way, even though the associations between QOL, SOC, and anxiety and depression in participants with breast cancer have been clarified in previous studies, specific supportive methods to increase QOL have not been indicated.

When we consider the QOL of our patients, it is necessary to consider their difficulties in daily life and subjective factors [9]. The World Health Organization (WHO) created the World Health Organization Disability Assessment Schedule 2.0 (WHODAS 2.0) as a comprehensive evaluation tool for gauging an individual’s activity limitations and participation restrictions. It also measures difficulties in daily life activities as a disability according to the International Classification of Functioning, Disability and Health (ICF) [9,10]. Among previous studies using WHODAS 2.0 to assess the QOL of survivors of breast cancer, Lourenço et al. [11] noted that patients with poor QOL experience many difficulties in daily life. Chachaj et al. [12] listed upper extremity pain, difficulty in arm movement, lymphedema, and prior chemotherapy as factors associated with decreased QOL and an increased WHODAS 2.0 score. The Chinese version [13] and the German version of WHODAS 2.0 [14] have been reported to be useful scales for assessing difficulties in the daily life of patients with breast cancer.

“Occupational dysfunction”, defined as a prominent factor affecting daily life difficulties, refers to the negative experiences individuals encounter when engaging in everyday activities. It represents a significant health-related issue that has primarily emerged within the realm of preventive occupational therapy [15]. This concept encompasses four domains in which individuals perceive limitations in their ability to perform activities of daily living [15]. These four domains are defined as follows: occupational imbalance, a loss of balance when performing daily activities [15,16]; occupational alienation, situations for which an individual’s inner needs related to daily activities are not met [14,15]; occupational deprivation, loss of opportunities to perform daily activities that are beyond the individual’s control [15,16]; and occupational marginalization, loss of an individual’s opportunities to perform desired daily activities [15,16]. Among healthcare workers, occupational dysfunction is a factor associated with psychological problems related to burnout syndrome, depression, and the stress response [17,18].

**Figure 1:**
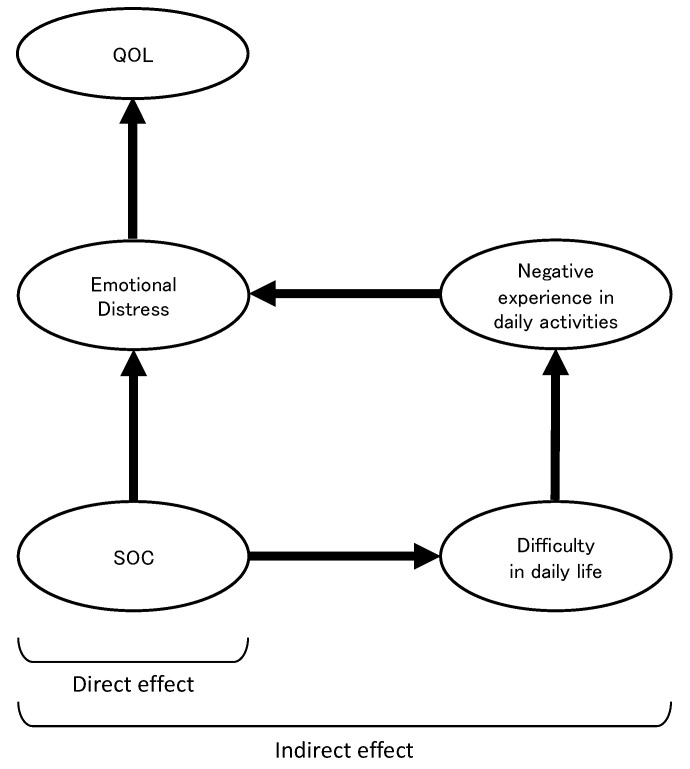
The hypothesis model. A hypothetical model of QOL in survivors of breast cancer includes the direct effect of “SOC” to “Emotional Distress” to “QOL” without mediating variables, and the indirect effect of “Difficulties in daily life” to “Negative experiences in daily activities” as mediating variables. QOL, quality of life; SOC, sense of coherence.

However, the structural relationship between difficulty in daily life and occupational dysfunction (negative experiences in daily activities) in the QOL of patients and survivors of breast cancer has not been investigated. Therefore, we developed a hypothetical model to construct a structural equation model of QOL based on difficulty in daily life, considering that difficulty in daily life and negative experiences in daily activities are included as variables in QOL (Figure 1). The hypothesized model was based on direct associations leading from “SOC” to “emotional distress (anxiety and depression)” and from “emotional distress” to “QOL”, which have already been shown in previous studies, and the additional structural relationships leading from “SOC” to “emotional distress” to “QOL” as mediated by “difficulty in daily life” and “negative experiences in daily activities.” Bayesian structural equation modeling (BSEM) was used to investigate the effectiveness of this model. If validated, this structural model could provide a basis for the development of support strategies to increase a survivor’s QOL by alleviating difficulties in daily life and improving negative experiences in daily activities. Thus, the purpose of this study was to clarify the structural relationship of QOL in survivors of breast cancer, including difficulty in daily life and negative experiences in daily activities as health-related indicators.

## 2. Materials and Methods

### 2.1. Participants

Participants in this multicenter cross-sectional study were survivors of breast cancer who belonged to 7 self-help groups (SHGs) and responded to recruitment requests between June and August 2021. Survivors of breast cancer who belonged to a SHG who agreed to participate in this study, and those who were eligible, were invited to participate in the study. The eligibility criteria were (i) at least 2 years after primary breast cancer surgery, (ii) 20 years old or older, and (iii) individuals who could understand the questionnaires and give their consent to the explanation of this study. Individuals with dementia, neuropsychiatric diseases, and physical disabilities before the first surgery for breast cancer were excluded. SHGs are voluntarily formed in small groups aiming at mutual assistance and achievement of specific goals [19], and breast cancer accounts for 78.7% of the SHGs formed for patients with cancer in Japan [20]. Participants were requested to answer the questionnaire via the web, and the results were provided to those who wished to receive them.

### 2.2. Procedure

General information such as sex, age, dominant hand, family structure, employment, and period of participation in SHGs and medical information such as diagnosis, date of surgery, age at the date of surgery, operation type, and stage classification were collected from the participants. The assessment used the Functional Assessment of Cancer Therapy-Breast (FACT-B), Hospital Anxiety and Depression Scale (HADS), 13-item version of SOC (SOC-13), WHODAS 2.0, and Classification and Assessment of Occupational Dysfunction (CAOD). Response to the surveys was requested by mail or web forms.

### 2.3. FACT-B

The FACT-B [21], a cancer-specific QOL scale, answers questions about one’s condition in the last seven days. FACT-B is composed of five domains: physical well-being (PWB), social/family well-being (SWB), emotional well-being (EWB), functional well-being (FWB), and breast cancer subscale (BCS). Participants were asked to answer 37 questions using a 5-point Likert-type scale, ranging from 0 (does not apply at all) to 4 (applies very well). The total score ranges from 0 to 148. The total score was calculated using the specified scoring method, with higher total scores indicating higher QOL. We used “Version 4 Japanese version” in this study.

### 2.4. HADS

The HADS is an anxiety and depression scale [22] answered considering one’s state in the past week. It consists of 7 items each that assess anxiety (HADS-A) and depression (HADS-D). Participants were asked to answer 14 questions on a 4-point Likert scale ranging from 0 to 3. A total score of ≥8 on the HADS-A (range: 0 to 21 points) was considered major anxiety, and a total score of ≥11 on the HADS-D (range: 0 to 21 points) was considered major depression.

### 2.5. SOC-13

The 13-item version of the 7-item SOC-13 [23] was used in this study. SOC-13 is scored from 1 to 7 points for each item. The range of scores was 13–91, and the general average was 54–58, with higher scores indicating better stress coping skills.

### 2.6. WHODAS 2.0

This study utilized the self-administered version of the WHODAS 2.0, which consists of 36 items [9,10]. This version assesses an individual’s functioning in six primary domains of life: (i) cognition (understanding and communication); (ii) mobility (ability to move and get around); (iii) self-care (ability to attend to personal hygiene, dressing and eating, and to live alone); (iv) getting along (ability to interact with other people); (v) life activities (ability to carry out responsibilities at home, work, and school); and (vi) participation in society (ability to engage in community, civil, and recreational activities). Participants were asked to respond to 36 questions, with six questions assigned to each domain. A 5-point Likert response scale ranging from “no problem” to “I can’t do anything at all” was employed. Scores for each domain and the total score were standardized. All scores (total and for each major life domain) range from 0 to 100, with higher scores indicating greater health-related difficulties in daily life.

### 2.7. CAOD

The CAOD is a tool utilized to assess occupational dysfunction, which refers to the negative experiences individuals face when they are unable to effectively perform activities of daily living [15]. Participants were asked to answer 16 questions using a 7-point scale ranging from “1 (disagree)” to “7 (agree)”. The total score on the CAOD ranged from 16 to 112, with higher scores indicating more severe negative experiences in daily activities.

### 2.8. Analysis

Confirmatory factor analysis was applied to each of the five assessment scales to measure their structural validity, and reliability coefficients were analyzed. The GFI was obtained from a model created with the corresponding sub-items influenced by each factor and covariance assumed among all factors. Each scale’s sub-items were considered based on the GFI, adjusted GFI (AGFI), and RMSEA, and Cronbach’s alpha coefficient was calculated to confirm the internal consistency of each scale. A GFI of >0.85 [24] and AGFI of >0.85 [24] were considered acceptable, as was an upper limit for RMSEA of <0.08 [25]. Based on previous studies, a hypothetical model was developed and verified using difficulties in daily life and negative experiences in daily activities as latent variables. The BSEM method was adopted due to the small sample size of this study [26]. Bayesian analysis is useful in that the prior distribution can be adjusted based on subjective and available information [27]. The prior distribution was assumed to be a uniform distribution with no specified range (model 1: principal analysis). In addition, sensitivity analyses with two different prior distribution settings were performed. The prior distributions of model 2 (sensitivity analysis) were assumed to be a uniform distribution with a specified range (range −1 to 1) for all path coefficients. The prior distributions of model 3 (sensitivity analysis) were analyzed as normal distributions [8,28] for QOL and emotional distress, SOC and emotional distress, respectively, while the other coefficients were analyzed as uniform distributions with no specified range due to a lack of information based on previous studies. If the causal model was significant, the multiplication of the path coefficient from “emotional distress” to “QOL” and from “SOC” to “emotional distress” was considered a direct effect on QOL, and the multiplication of the path coefficient from “SOC” to “difficulty in daily life”, from “difficulty in daily life” to “negative experiences in daily activities”, and from “negative experiences in daily activities” to “emotional distress” was considered an indirect effect on QOL. BSEM estimation was conducted using the Markov chain Monte Carlo method. The goodness of fit for the model was evaluated using the posterior prediction method and the posterior predictive *p*-value (PPP), with a PPP >0.10 indicating a good fit of the model [29]. Furthermore, path coefficients and their corresponding 95% confidence intervals (CIs) between the latent variables in the model were analyzed. A path coefficient was considered statistically significant if the 95% CIs did not include zero. The model was run with a total of 100,000 sampling times, and convergence of the algorithm was indicated by a convergence statistic set at <1.002.

Statistical significance was determined using a *p*-value of <0.05. Statistical analyses were performed using the following software: SPSS Statistics 27 (IBM, New York, NY, USA), SPSS Amos ver. 25.0 (IBM, New York, NY, USA), and R (version 4.1.2).

### 2.9. Ethics Statement

This study was performed in line with the principles of the Declaration of Helsinki. Approval was obtained from the Ethics Committee of Kitasato University School of Allied Health Sciences (date: 26 April 2021/Approval No.: 2019-030-200) as well as from each participating SHG. The participants were provided with a clear explanation of the study’s purpose and content, and informed consent was obtained from all individual participants included in this study.

## 3. Results

### 3.1. Participant Characteristics

The characteristics of the participants are shown in Table 1. The participants comprised 73 survivors of breast cancer with an average age of 62.4 ± 8.6 years. It had been 13.1 ± 7.6 years since the first surgery, and about half of the participants were currently employed.

The scores for each assessment scale are shown in Table 2. The average score of FACT-B was relatively high, but that of SWB was lower than the other domains. The average scores of HADS-A and D were lower than the respective cutoff values and were not judged as major anxiety or major depression. The average score of SOC-13 was higher than the general average of 54–58, indicating that most participants had high stress coping skills. The WHODAS 2.0 scores indicated that the patients experienced difficulties in participation in society, getting along, and life activities. All domains of the CAOD were lower than the median of the score range.

### 3.2. Relationship between Each Assessment Scale

A confirmatory factor analysis was performed on the sub-items of each assessment scale. The goodness-of-fit indices, including GFI, AGFI, and RMSEA were as follows: FACT-B; GFI = 0.980, AGFI = 0.901, RMSEA = 0.057, HADS; GFI = 0.896, AGFI = 0.835, RMSEA = 0.000, SOC-13; GFI = 0.901, AGFI = 0.847, RMSEA = 0.000, WHODAS 2.0; GFI = 0.777, AGFI = 0.702, RMSEA = 0.043, CAOD; GFI = 0.875, AGFI = 0.797, RMSEA = 0.044. These values confirm that the original factor structures of the assessment scales were supported by the data. Cronbach’s alpha for each scale was 0.805 for FACT-B, 0.928 for HADS, 0.864 for SOC-13, 0.902 for WHODAS 2.0, and 0.928 for CAOD.

### 3.3. Structural Relationship of QOL, Emotional Distress, SOC, Difficulty in Daily Life, and Negative Experiences in Daily Activities

**Figure 2:**
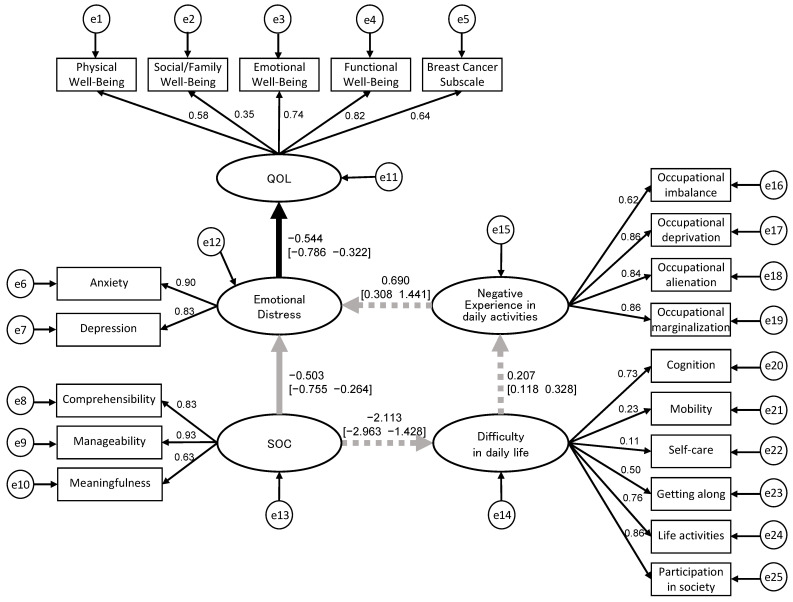
Structural relationship between the recovery process and difficulties in daily life mediated by negative experiences in daily activities using BSEM (model 1: principal analysis). The values in this figure indicate the standardized path coefficients (95% CI). The prior distribution was assumed to be a uniform distribution with no specified range. PPP = 0.20; model fit is good. Direct effect (the solid gray and black lines) = 0.274, “SOC”—“emotional distress”—“QOL”. Indirect effect (the dotted gray and black lines) = 0.163, “SOC”—“difficulty in life”—“negative experiences in daily activities”—“emotional distress”—“QOL”. QOL, quality of life; SOC, sense of coherence; PPP, posterior predictive *p*-value.

A hypothetical model of causality in QOL was developed (Figure 1), and BSEM was employed to analyze the model. The latent variables in the model included “QOL”, “emotional distress”, “SOC”, “difficulty in daily life”, and “negative experiences in daily activities”, while the observed variables were the scores on the sub-items of each assessment scale. The Bayesian estimation demonstrated a stable convergence statistic, with values ranging from 1.000 to 1.002. This indicates reliable estimation results. The PPP was 0.20, which was better than 0.10, indicating a satisfactory fit of the model. The standardized path coefficients [95% CI] between each latent variable in the BSEM were −0.503 [−0.755, −0.264] from “SOC” to “emotional distress” and −0.544 [−0.786, −0.322] from “emotional distress” to “QOL” as the direct effects, both significant. The direct effect from “SOC” to “QOL” via “emotional distress” was 0.274 (−0.503 × −0.544). The indirect effects from “SOC” to “difficulty in daily life” was −2.113 [−2.963, −1.428], from “difficulty in daily life” to “negative experiences in daily activities” was 0.207 [0.118, 0.328], and from “negative experiences in daily activities” to “emotional distress” was 0.690 [0.308, 1.441], all of which were significant. The indirect effect from “SOC” to “QOL” mediated by “difficulties in daily life” and “negative experiences in daily activities” was 0.164 (−2.113 × 0.207 × 0.690 × −0.544) (Figure 2). The BSEM was conducted as sensitivity analysis. The PPP was 0.77 for model 2 and 0.65 for model 3, indicating a satisfactory fit of the model. The direct and indirect effects for model 2 were 0.311 and 0.183, respectively, and for model 3 were 0.309 and 0.158, respectively (Supplement Table S1, Supplement Figures S1 and S2).

## 4. Discussion

In addition to the direct association supporting the conventional finding that improvement in SOC, which is the individual’s ability to cope with stress, leads to improvement in QOL mediated by emotional distress, an indirect association was indicated showing that a decrease in difficulty in daily life and impaired negative experiences in daily activities leads to improvement in QOL mediated by emotional distress for the structural relationship of QOL in survivors of breast cancer. The direct association from “SOC” to “QOL” via “emotional distress” supported the findings of previous studies [6,7,8]. The direct effect of this study was 0.274, which was almost comparable to the results of previous studies conducted in other cultures, such as by Zamanian et al. [30] and Rohani et al. [31]. According to Antonovsky [5], SOC plays a crucial role in enabling individuals or groups to effectively cope with stressors and life crises while also tapping into their growth potential. SOC is developed through positive environments and life experiences, typically until around the age of 30. Subsequent life experiences can change SOC, and SOC can be improved by intervention programs based on SOC theory [32]. Therefore, for persons with low SOC, this intervention program may increase their SOC, improve their levels of anxiety and depression, and thus improve their QOL. However, the indirect effect was 0.163. Comparing the path coefficients in previous studies using SEM, the path coefficient between WHODAS 2.0 and SOC-13 was −0.178 in the Moen et al. [33] study of rehabilitation patients and was smaller than the path coefficient between variables in the present study. In a study of healthcare workers by Teraoka and Kyougoku [18], the path coefficient between CAOD and Depression was 0.745, which was similar to the path coefficient in the present study. The path coefficient of WHODAS 2.0 and CAOD was 0.598 in the Watanabe et al. study [34] of severe and persistent mental illness (SPMI), whereas that in the present study was smaller. The previous studies have used different participants, and to our knowledge, no previous studies have verified these structural relationships in participants with breast cancer, making the present study the first to do so. Although the indirect effect in this study was smaller than the direct effect, the possibility was indicated that QOL could be improved by providing support using the indirect effect to “emotional distress” mediated by “difficulty in daily life” and “negative experiences in daily activities.” The reason for the lack of convergence of the BSEM with the addition of age is thought to be due to the limited age range of the study participants and to the bias of the participants because they were recruited from SHGs. As mentioned previously [32], although SOC is established by around age 30 and may change thereafter, age bias as a disease characteristic was undeniable.

Establishing psychosocial support strategies in the context of survivorship of breast cancer is important for improving QOL [35]. As a direct effect, QOL can be enhanced by improving anxiety and depression, which can be done with pharmacotherapy, exercise therapy [36], and psychotherapy [37]. The Oncology Nursing Society (ONS) identified self-management as a priority topic for 2014–2018 [38] and stated that it is important to support people in self-managing the symptoms and effects of cancer. Yamanaka et al. [39] also reported that cancer patients need to self-manage their symptoms after outpatient treatment when opportunities for direct intervention by healthcare providers decrease and that this has been shown to improve QOL. However, these are organic aspects based on psychological indices, and various factors are involved. Further, it is difficult to use these coping methods by oneself unless the community and environment for implementing these methods are in place. Therefore, we found it necessary to construct a model that can approach survivors’ daily life and clarify the extent of the effect. Regarding indirect effects, in a report of the BSEM for SPMI, it was clarified that WHODAS 2.0 was involved in the improvement of recovery of patients with SPMI using CAOD as a mediating variable [34]. This indicates the importance of capturing negative experiences in daily activities as a meaning of difficulty in daily life rather than only the presence or absence of difficulty in daily life. Thus, CAOD is said to be a subjective factor in difficulty in daily life, and careful assessment of CAOD and intervention for problems in the four domains may improve anxiety, depression, and QOL as well. The model in the present study provided valid information because it suggested that the provision of such information and practice by survivors may lead to improved QOL.

There are many SHGs for breast cancer patients in Japan, although they vary widely in size and activities. Therefore, the participants in this study may not be considered general survivors. The participants in this study were survivors of primary breast cancer surgery for more than 2 years after surgery in the Japanese culture. The majority were survivors who had completed postoperative therapy, but some were still undergoing treatment at the time of the assessments. In addition, participants were included regardless of their experience with cancer recurrence. Therefore, different cultures, treatment statuses, and experiences with cancer recurrence may lead to differences in the results of the assessments. There were selection biases that need to be carefully considered to determine whether the insights gained from this model can be generally applied. In addition, an assessment of subjective indicators needs to control for mood as a potential confounding factor [40]. Even though all of the assessment scales used in this study were subjective and self-administered scales, they did not assess or adjust for the participants’ mood states, such as depression. Furthermore, Alagizy et al. [41] reported that postoperative patients with breast cancer who were married had higher moderate or severe anxiety and depression than breast cancer patients who were single, and, among patients with breast cancer, those who were unemployed had significantly higher anxiety than those who were employed. Thus, the analysis may need to take into account the different post-treatment periods and environments of the participants. However, to the best of our knowledge, the results of this study represent a new model of care strategy that should help to identify specific strategies to improve QOL in terms of difficulty in daily life. In addition, the relationship between difficulty in daily life and QOL in the immediate postoperative period may show a different degree of efficacy. The results of this study can be useful as basic comparative information because the model was constructed and validated based on standardized rating scales.

## 5. Conclusions

The present empirical research has shown a structural relationship between QOL mediated by difficulties in daily life and negative experiences in daily activities in survivors of breast cancer. The present model indicates that difficulty in daily life and negative experiences in daily activities are mediating variables and that support not only directly decreases difficulty in daily life but also addresses the negative experiences in daily activities as a subjective aspect that arises from difficulty in daily life that may improve QOL. The influence of difficulties in daily life, which were not revealed by the support for anxiety, depression, and SOC shown in previous studies, was clarified. Therefore, it is important to focus on the difficulties in daily life and negative experiences in daily activities of individual survivors of breast cancer and provide support to improve them. These direct and indirect approaches to addressing difficulties in daily life are very significant because they can provide a basis for the development of support strategies to increase QOL. Expected research in the future would be based on a longitudinal study to examine whether difficulty in daily life could affect changes in QOL.

## Figures and Tables

**Table 1:** Characteristics of the participants.

Characteristic	Value
Participants [*n*]	73
Male	0
Female	73
Age [*years (range)*]	62.4 ± 8.6 (44–80)
Period of education [*years (range)*]	14.3 ± 2.1 (10–22)
Marital status [*n*]	
Married	53 (72.6%)
Divorced	5 (6.9%)
Not married	9 (12.3%)
Bereaved	6 (8.2%)
Family structure [*n*]	
Living together	62 (84.9%)
Separate	11 (15.1%)
Type of surgery [*n*]	
Mastectomy	47 (64.4%)
Breast-conserving surgery	16 (21.9%)
Lumpectomy	9 (12.3%)
Unknown	1 (1.4%)
Clinical stage [*n*]	
stage 0	2 (2.7%)
stage I	26 (35.6%)
stage II	27 (37.1%)
stage III	7 (9.6%)
stage IV	2 (2.7%)
Unknown	9 (12.3%)
Duration from first surgery ^a^ [years (range)]	13.1 ± 7.6 (5.0–36.4)
Treatment of post-surgery [*n*]	
Chemotherapy	39 (53.4%)
Radiotherapy	38 (52.1%)
Hormone therapy	55 (75.3%)
Others	16 (21.9%)
Current job status [*n*]	
Working	35 (47.9%)
Not working	38 (52.1%)

Values are mean ± standard deviation (range), n (percentage). ^a^ *n* = 71 (due to inclusion of two unknown data).

**Table 2:** Assessment scores.

Assessment (Range of Score)	Score (Range)
FACT-B (0–148)	100.5 ± 19.2 (60–137)
Physical Well-Being (0–28)	23.3 ± 5.2 (8–28)
Social/Family Well-Being (0–28)	15.8 ± 6.6 (2–28)
Emotional Well-Being (0–24)	17.7 ± 4.5 (7–24)
Functional Well-Being (0–28)	19.7 ± 5.5 (5–28)
Breast Cancer Subscale (0–40)	24.1 ± 5.9 (2–33)
HADS	
HADS-A (0–21)	6.6 ± 4.8 (0–20)
HADS-D (0–21)	6.4 ± 4.1 (0–19)
SOC-13	
Total (13–91)	62.1 ± 12.9 (27–87)
Comprehensibility (5–35)	23.3 ± 6.2 (7–34)
Manageability (4–28)	18.1 ± 4.8 (7–28)
Meaningfulness (4–28)	20.8 ± 3.8 (10–28)
WHODAS 2.0 (0–100)	
Total score	14.1 ± 10.6 (0–40)
Cognition	9.5 ± 14.3 (0–80)
Mobility	9.7 ± 13.3 (0–56.3)
Self-care	1.5 ± 5.2 (0–30)
Getting along	20.7 ± 18.2 (0–75)
Life activities	18.6 ± 20.8 (0–70)
Participation in society	21.7 ± 17.9 (0–58.3)
CAOD	
Total score (16–112)	44.1 ± 19.3 (16–95)
Occupational imbalance (4–28)	11.3 ± 6.2 (4–26)
Occupational deprivation (3–21)	9.9 ± 5.2 (3–21)
Occupational alienation (3–21)	8.7 ± 4.5 (3–18)
Occupational marginalization (6–42)	14.2 ± 6.9 (6–35)

Scores are mean ± SD (range). FACT-B, Functional Assessment of Cancer Therapy-Breast; HADS, Hospital Anxiety and Depression Scale; SOC, sense of coherence; WHODAS 2.0, WHO Disability Assessment Schedule 2.0; CAOD, Classification Assessment of Occupational Dysfunction.

## Data Availability

The data presented in this study are available on request from the corresponding author.

## Supplementary Materials

The following supporting information can be downloaded at https://www.mdpi.com/article/10.3390/healthcare11142082/s1, Figure S1: model 2 (Sensitive analysis): Structural relationship between the recovery process and difficulties in daily life mediated by negative experiences in daily activities using BSEM; Figure S2: model 3 (Sensitivity analysis): Structural relationship between the recovery process and difficulties in daily life mediated by negative experiences in daily activities using BSEM; Table S1: Results of sensitivity analyses.
