# Supplementary material for: Estimation of a Structural Equation Modeling of Quality of Life Mediated by Difficulty in Daily Life in Survivors of Breast Cancer
Source: Healthcare (Basel). 2023 Jul 21;11(14):2082. doi: 10.3390/healthcare11142082 (PMC10379596; doi:10.3390/healthcare11142082)
Supplement: Supplementary file 1 [file healthcare-11-02082-s001.zip › Table S1.pdf]

**Supplement Table S1** Results of sensitivity analyses

| | analysis | direct effect | indirect effect |
|---|---|---|---|
| model 1 | (principal analysis) | 0.274 | 0.163 |
| model 2 | (sensitivity analysis) | 0.309 | 0.158 |
| model 3 | (sensitivity analysis) | 0.307 | 0.182 |
