# Supplementary figures and images for: Estimation of a Structural Equation Modeling of Quality of Life Mediated by Difficulty in Daily Life in Survivors of Breast Cancer
Source: Healthcare (Basel). 2023 Jul 21;11(14):2082. doi: 10.3390/healthcare11142082 (PMC10379596; doi:10.3390/healthcare11142082)

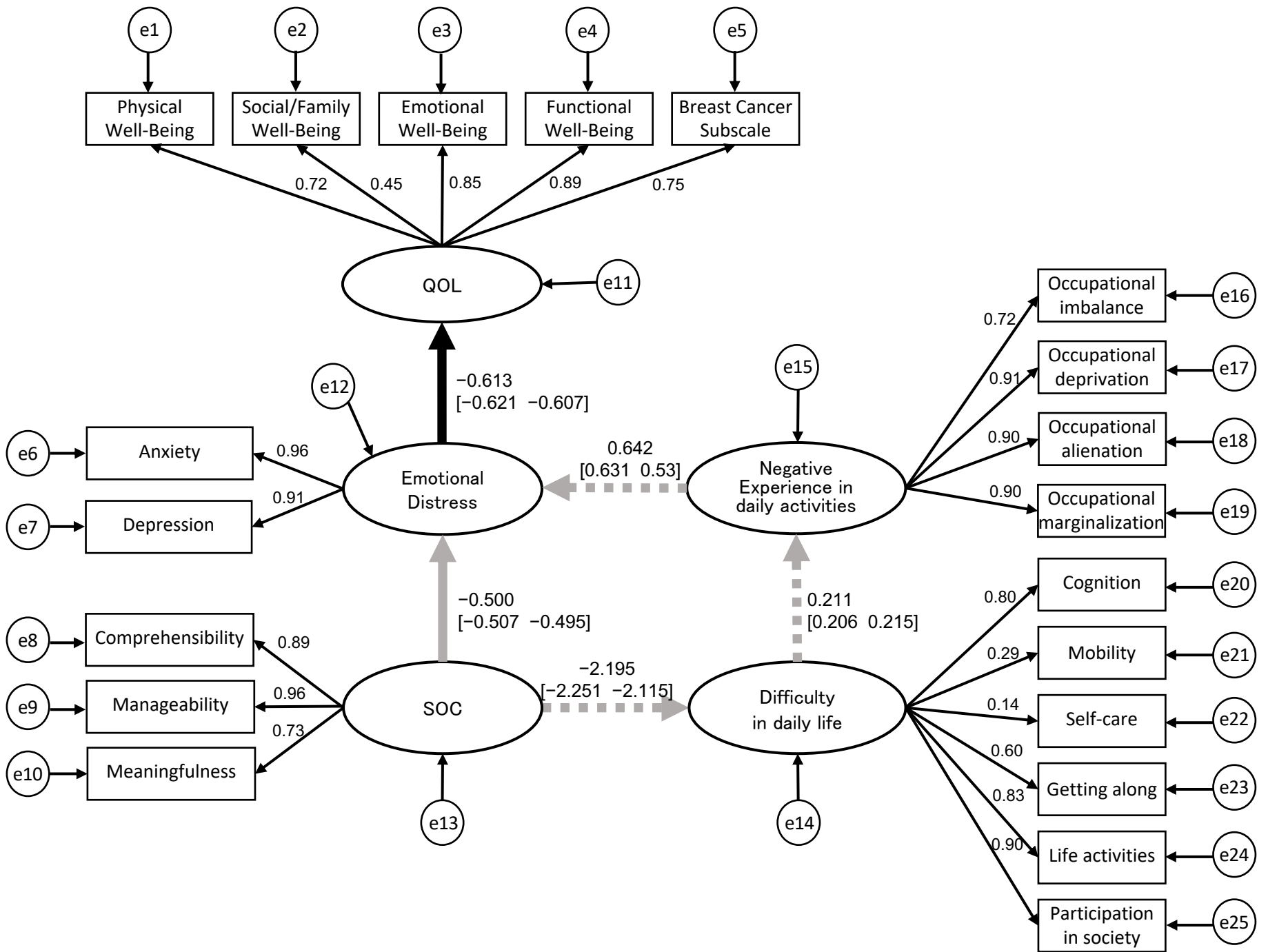

Supplement: Supplementary file 1 [file healthcare-11-02082-s001.zip › Figure S1.pdf]

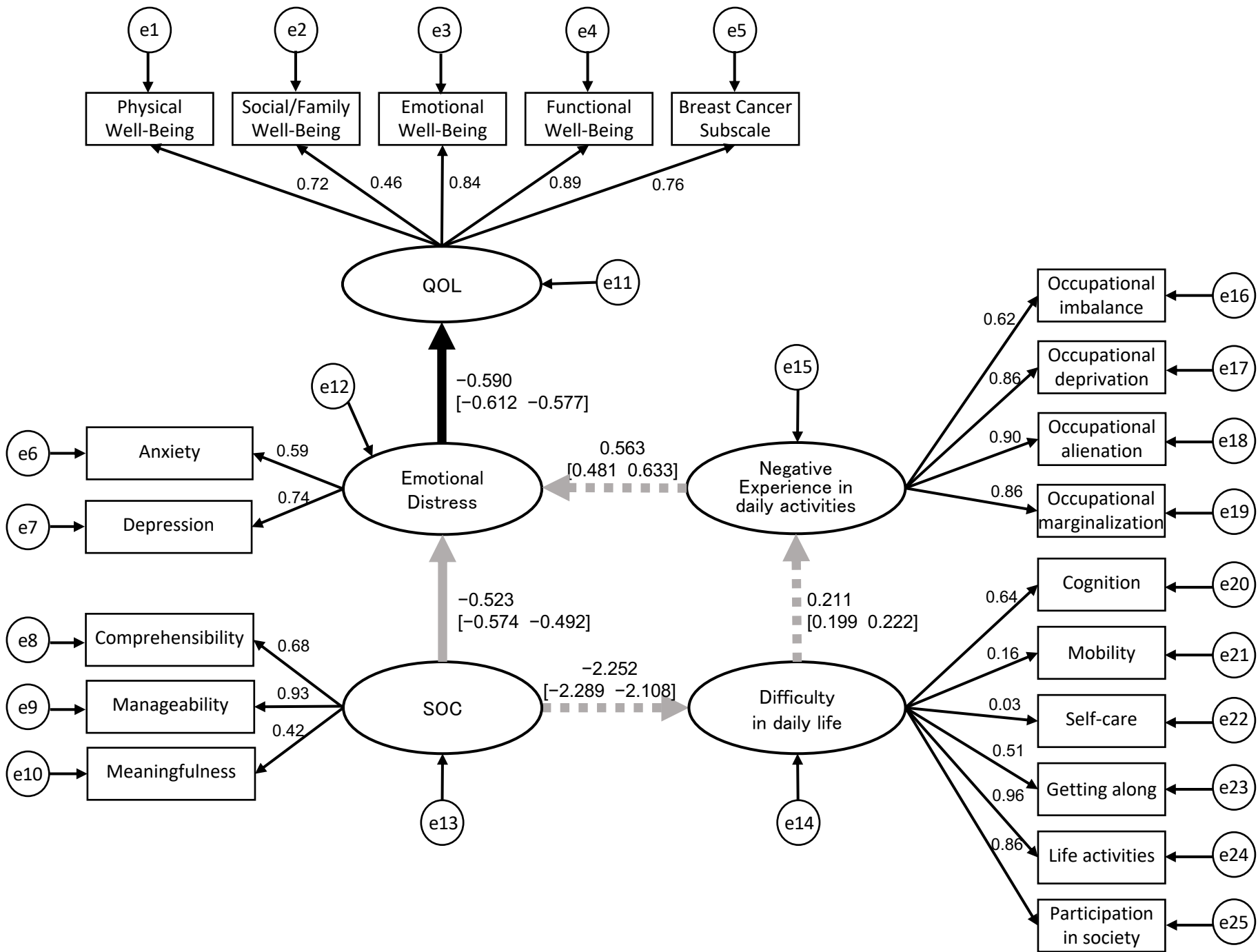

Supplement: Supplementary file 1 [file healthcare-11-02082-s001.zip › Figure S2.pdf]
